# An innovative approach to close large mucosal defects post-endoscopic submucosal sissection: threads combined with endoclips

**DOI:** 10.1055/a-2535-1748

**Published:** 2025-03-03

**Authors:** Jia Xu, Weixing Yang, Zhongqiong Wang, Muhan Lü, Xiaowei Tang

**Affiliations:** 1 556508 Department of Gastroenterology, The Affiliated Hospital of Southwest Medical University, Luzhou, China

Endoscopic submucosal dissection (ESD) is an effective minimally invasive treatment for early gastrointestinal cancers, offering benefits such as larger resection areas and high cure rates 1 . However, post-ESD procedures often leave large mucosal defects, creating significant complications and obstacles for clinical closure 2 3 . Endoclips are commonly used for closure, but they can be ineffective for large defects where direct closure is difficult 4 .

This report presents a novel closure technique demonstrated through a specific case ( Video 1 ). The patient was a 75-year-old man who underwent ESD for a rectal tumor. After the procedure, the remaining large mucosal defect was too wide for direct closure using standard endoclips ( Fig. 1 ). Our approach was as follows. A cotton thread was first tied to an endoclip, which was then fixed to the upper left edge of the wound, with the other end of the thread extending out of the anus. This was repeated to secure a second endoclip with a thread at the upper right edge of the wound ( Fig. 2 ). Pulling the threads externally reduced the original defect width to a size manageable by endoclips. Subsequently, we applied additional clips to close the upper and lower wound edges at the center ( Fig. 3 ) and then progressively used clips from the center outward to close the wound entirely. Finally, we concluded by cauterizing the cotton threads ( Fig. 4 ). Based on this procedure, we developed a novel device consisting of cotton threads and endoclips for post-ESD defects ( Fig. 5 ).

**Fig. 1:**
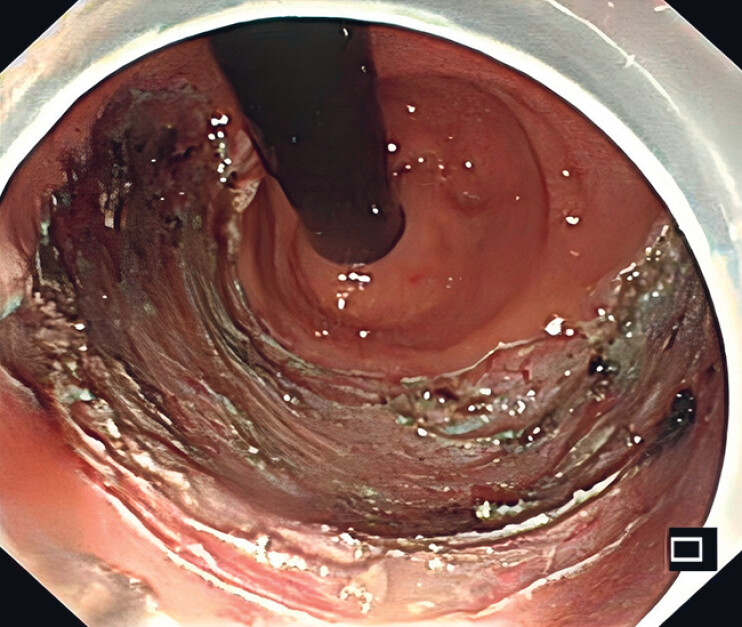
The large mucosal defect left after endoscopic submucosal dissection.

**Fig. 2:**
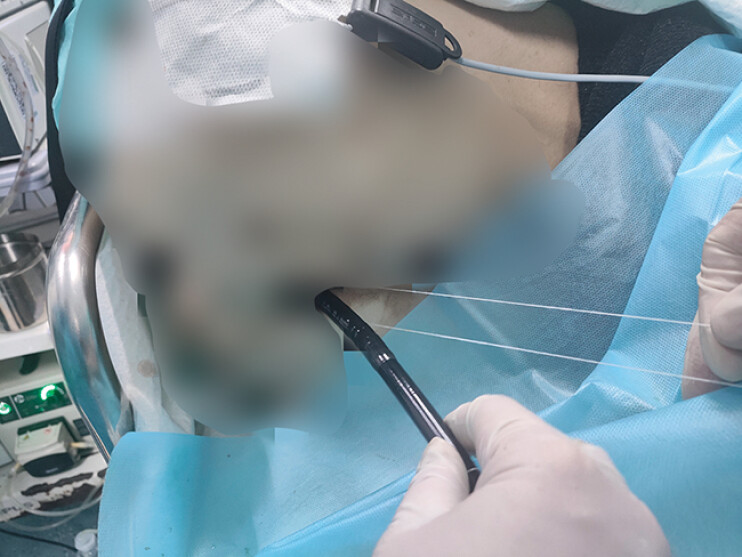
Two endoclips securing the cotton threads were fixed to the upper left and right edges of the defect.

**Fig. 3:**
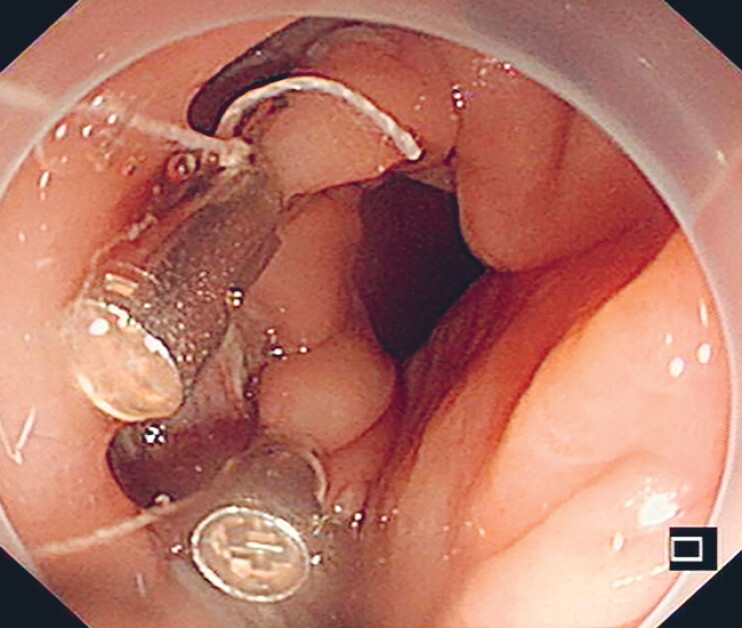
Pulling the threads externally narrowed the original defect width to a size suitable for closure with endoclips. A clip was then applied to close the upper and lower edges of the wound at the center.

**Fig. 4:**
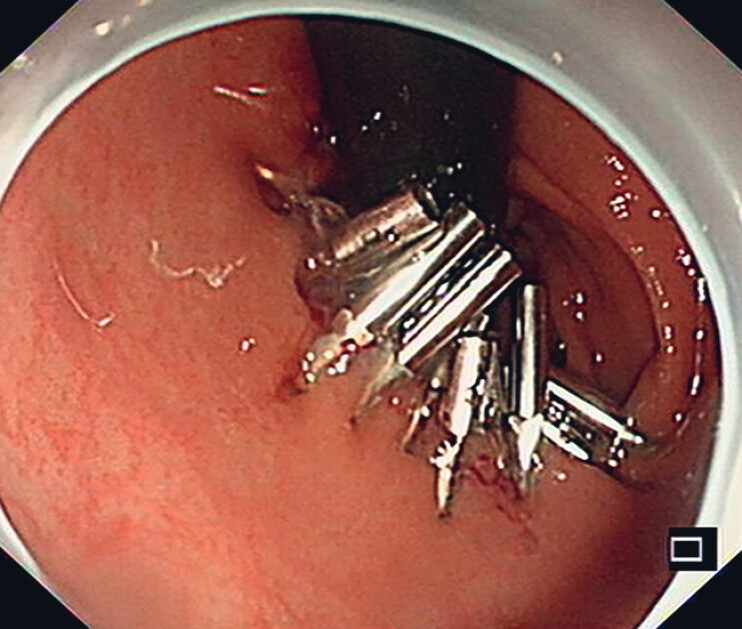
We sequentially closed the defect from the center to the sides using endoclips and then cauterized to cut the cotton threads.

**Fig. 5:**
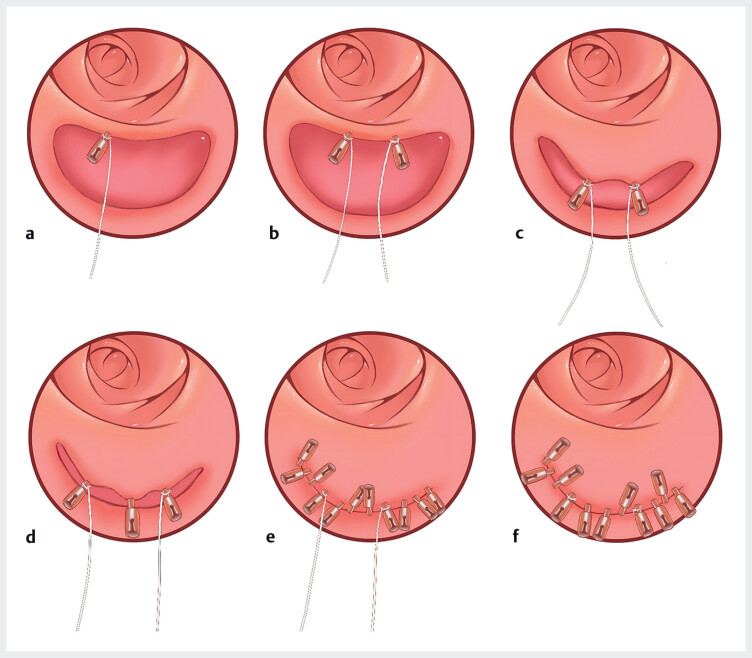
Schematic diagram for effectively closing a large mucosal defect following endoscopic submucosal dissection using cotton threads combined with endoclips. **a** Loop a cotton thread around one endoclip and secure the clip to the upper left edge of the wound. **b** Secure another looped endoclip to the upper right edge of the wound. **c** Pulling the thread externally reduces the original wound width to a size that can be closed with endoclips. **d** A clip is then applied to close the upper and lower edges of the wound at the center. **e** Apply endoclips to close the wound from the center toward both sides. **f** Cut off the threads.

Using cotton threads combined with endoclips to effectively close a large mucosal defect following endoscopic submucosal dissection.Video 1

This approach enables large mucosal defects to be closed in a minimally invasive manner and enhances operational efficiency. Additionally, the technique is straightforward, easily mastered, and cost-saving, providing a valuable new option for post-ESD large defect closures.

Endoscopy_UCTN_Code_TTT_1AQ_2AK

## Notes

Endoscopy E-Videos https://eref.thieme.de/e-videos
*E-Videos* is an open access online section of the journal *Endoscopy* , reporting on interesting cases and new techniques in gastroenterological endoscopy. All papers include a high-quality video and are published with a Creative Commons CC-BY license. Endoscopy E-Videos qualify for HINARI discounts and waivers and eligibility is automatically checked during the submission process. We grant 100% waivers to articles whose corresponding authors are based in Group A countries and 50% waivers to those who are based in Group B countries as classified by Research4Life (see: https://www.research4life.org/access/eligibility/ ). This section has its own submission website at https://mc.manuscriptcentral.com/e-videos .
